# The ‘Positive Effect’ Is Present in Older Chinese Adults: Evidence from an Eye Tracking Study

**DOI:** 10.1371/journal.pone.0121372

**Published:** 2015-04-16

**Authors:** Jingxin Wang, Liyuan He, Liping Jia, Jing Tian, Valerie Benson

**Affiliations:** 1 Academy of Psychology and Behaviour, Tianjin Normal University, Tianjin, China; 2 Centre for Visual Cognition, School of Psychology, University of Southampton, Southampton, United Kingdom; Catholic University of Sacred Heart of Rome, ITALY

## Abstract

The 'Positive Effect' is defined as the phenomenon of preferential cognitive processing of positive affective information, and avoidance or dismissal of negative affective information in the social environment. The ‘Positive Effect’ is found for older people compared with younger people in western societies and is believed to reflect a preference for positive emotional regulation in older adults. It is not known whether such an effect is Universal, and in East Asian cultures, there is a highly controversial debate concerning this question. In the current experiment we explored whether Chinese older participants showed a 'Positive Effect' when they inspected picture pairs that were either a positive or a negative picture presented with a neutral picture, or a positive and negative picture paired together. The results indicated that both groups of participants showed an attentional bias to both pleasant (more processing of) and unpleasant pictures (initial orienting to) when these were paired with neutral pictures. When pleasant and unpleasant pictures were paired together both groups showed an initial orientation bias for the pleasant picture, but the older participants showed this bias for initial orienting and increased processing measures, providing evidence of a ‘Positive Effect’ in older Chinese adults.

## Introduction

Subjective well-being is an important comprehensive index to measure individual life quality, and is defined as the overall evaluation for the quality of the life according to the standard which individual’s set themselves. Life satisfaction and emotional experience are two basic elements of subjective well-being [1]. There are reasons to believe that subjective well-being should decline as people get older since both physical health and cognitive abilities decline as the amount of lifetime remaining decreases [2]. Yet, the frequency of experiencing negative emotions also decreases throughout most of adulthood, and levels off around the age of 60 [3] resulting in the 'Positive Effect'. The 'Positive Effect' refers to the preferential cognitive processing of positive stimuli relative to negative stimuli as people get older. This effect remains largely stable across the older adult lifetime, although some studies show modest increases [4] or slight decreases [5] with age. Accordingly, older people should feel happier than the younger people.

In recent years, most studies on the ‘Positive Effect’ have been conducted in Western countries, especially in the Euro-American region. Researchers have investigated the ‘Positive Effect’ on attention and memory. For example, Charles, Mather and Carstensen [6] found a ‘Positive Effect’ in older people using recognition and recall tests after presenting positive, neutral, and negative pictures to younger people (18–29 years), adults (41–53 years), and older people (65–85 years). Although the general performance decreases with age, the ratio of positive pictures to negative pictures memory performance increases with age, implying that as one gets older they are more likely to remember more positive pictures. In another study Mather and Carstensen [7], examined age differences in attention and memory for emotional faces using a dot-probe paradigm. They found that older people responded faster when the dot probe was presented on the side where a neutral face had been presented, compared to when a negative face had been presented there. Moreover, older participants remembered the positive faces better than the negative faces. In a later study, Isaacowitz, Wadlinger, Goren, and Wilson [8] found a ‘Positive Effect’ among older people when investigating how adults’ attended to synthetic faces with emotional or neutral expressions using eye tracking methodology. Younger participants showed an attentional preference toward faces expressing fear while older participants preferred positive (e.g., happy) faces and avioded attending to negative (e.g., angry) faces. There are many other studies investigating the ‘Positive Effect’ in Western participants [9–14], and the effect has been found consistently in older adults. The socioemotional selectivity theory (SST), originally put forward by Carstensen in 1999 [15], explained the effect as resulting from the difference between emotional goal related motivation, and knowledge goal related motivation, when processing affective information. Later [16, 17], the SST was extended to explain the increased preference for positive to negative material in older adults. The argument stated that as people age there are changes in goals that increasingly favour emotional well-being. The theory predicts that older, compared to younger, people will prefer to process positive affective material, because of the limited time they have left to live. This results in a motivation to restrict any processing, and thus any impact, of negative material. In contrast younger adults attend to negative information in order to pursue knowledge related goals in their quest to acquire new information to prepare for their future.

It is not known whether such a ‘Positive Effect’ in older people is universal and research into the ‘Positive Effect’ in East Asian cultures has to date been rather limited with inconsistent findings. There may be cultural differences relating to emotional processing that could impact on whether all older people show a ‘Positive Effect’ [18, 19]. It is known that culture plays an important role on human behavior and impacts on eye movements. For example, in an eye-tracking experiment (Chua, Boland, & Nisbett, 2005) [20], Chinese and American graduate students were asked to look at a series of pictures with a single focal animal or object and a richly detailed background, rate how much they liked each picture, and then complete an object recognition test, a paradigm similar to the experiment of Masuda and Nisbett (2001) [21]. The results showed that there were cross-cultural differences of the eye movement patterns between the Chinese and the Americans. In sum, the Chinese made more background fixations than the Americans, and compared to the Chinese, the Americans had substantially longer fixations on objects than on backgrounds. Chua et al. interpreted their findings to suggest that people from different cultural backgrounds see the world differently. Indeed, Western cultures are individualistic and people are encouraged to publicly express themselves and become independent. They cultivate a sense of pride and optimism at an individual level, and, compared with East Asians living in collective cultures, Western people who believe in individualism, have preferential cognitive processing of positive stimuli with high arousal levels [22]. In contrast, East Asians praise highly collective cultures where the focus is on interdependence between individuals. In this type of culture people are educated to adapt to the collective and it is viewed as important to try to get along with others in harmony. People consider that the individual is part of the collective, and unity and solidarity are seen as being more important than individualism. Under this kind of culture, endurance and concession are inevitable. Therefore the suppression of some positive emotions is important to adapt to the collective in an Eastern culture. In such a culture the ‘Positive Effect’ that is observed in older adults in Western cultures, may either be reduced or absent. In a recent study (Reed, Chan & Mikesl, 2014) [23], a systematic meta-analysis was conducted for 100 empirical studies of the ‘Positive Effect’. The findings indicated that the ‘Positive Effect’ for older adults is reliable but may be moderated by methodological (such as constrained vs unconstrained cognitive processing) and sample characteristics (wider vs narrower age comparisons). However the authors also pointed to a limitation of their meta-analysis, which is that it could not examine cross-cultural differences leading to the view that more cross-cutural work is needed to investigate the inconsistent results for the ‘Positive Effect’ across cultures.

In a replication of Isaacowitz et al.’s study [8], Fung and colleagues did not find evidence of a ‘Positive Effect’ in older Chinese attentional preferences for angry and fearful faces. Additionally, no attentional bias was found in younger participants for any emotional or neutral faces. The authors argued that the differences between their study and the fidings of Isaacowitz et al.’s study [8], resulted from cultural differences between Americans and Eastern participants, as negative information is as important as positive information in an Eastern culture in order to adapt to an East Asian society. This finding is in contrast with Kwon, Scheibe, Samanez-Larkin and Tsai [24], who replicated the finding of a ‘Positive Effect’ for a picture memory task in Koreans, who are also East Asians. In that study the participants were asked to perform recognition and recall tests and to give valence ratings after viewing emotional images. Older Koreans had better memory for positive images than for negative images.

The reported findings to date are therefore inconsistent in relation as to whether or not a ‘Positive Effect’ is observed in older people in East Asian cultures. It could be argued that some of the inconsistencies might reflect the nature of the materials used or differences in the task requirements e.g. Fung et al. [18] and Isaacowitz et al.’s study [8]. In the current study we investigated the ‘Positive Effect’ in Chinese participants, by recording eye movements and examining whether there was evidence of attentional preferences to orient to, and process, positive emotional pictures.

It is well established that there is a link between eye movements and on-line cognitive processing for a range of tasks [25], and, it is also known that attention is highly associated with eye movement patterns. For example, eye movements can show how attention is deployed [26, 27] and that where people look, and for how long, can be driven by task instruction as well as stimulus properties.

In Eastern cultures suppression of positive self-expression and a focus on self-criticism could lead to preferential attention to negative rather than positive information, and this would not be deemed to alter throughout the lifetime. Such cultural differences between East and West mean that Chinese participants, who have grown up to be part of a collective interdependent culture, may assign the same value to negative and positive emotions when they are both younger and older, whereas Western participants should assign less value to negative emotions as they get older. If that is the case we hypothesis that the ‘Positive Effect’ will be either smaller, or absent, in older Chinese adults. If it is the case that the ‘Positive Effect’ results from emotional self regulation [25], and that this type of emotional strategy develops in all older people—then cultural differences should be irrelevant and the hypothesis here would be that older Chinese adults should show a ‘Positive Effect’.

## Method

### Ethics statement

This study was approved by the Institutional Review Board of Tianjin Normal University, and every participant provided written informed consent before taking part in the experiment.

### Participants

The sample included 27 younger adults (18 women, 9 men, mean age = 22.9, range = 19–27 years, education age = 12–18 years, mean age = 15.8 years) and 26 older adults (18women, 8 men, mean age = 64.4 years, range = 60–74 years, education age = 6-15years, mean age = 11.6 years) residing in Tianjin, China. Younger participants were recruited from a local university. Older participants were recruited from residential districts nearby. All participants were right-handed, had normal or corrected-to-normal vision and had no neurological or mental disorders. No participant had taken part in similar experiments, and all participants were paid fifty yuan for volunteering.

Before taking part in the experiment demographic data and scores on the Positive Affect and Negative Affect Scale (PANAS) (developed by Watson, Clark, & Tellegen, 1988 and revised by Qiu et al. 2008 which is adapted for Chinese)[28, 29] were collected. We also tested participants on the vocabulary and numerical sub-tests of the Wechsler Intelligence Test. The results from these, and other participant demographics are shown in Table 1. The difference between the groups in the numerical scores is likely to reflect the difference in years of education. It is however not anticipated that these differences should impact upon how emotional information is attended to and processed.

**Table 1:** Demographic data and Cognition Tests (Means) for all Participants.

	Younger	Older	*t*
**Sample Size**	27	26	—
**Male**	9	8	—
**Female**	18	18	—
**Age Range**	19–27	60–74	—
**Mean Age**	22.9	64.4	—
**Years of Education**	15.8	11.6	-6.33*
**Emotion Tests (PANAS)**	11.37	13.65	1.17
**Vocabulary Tests**	66.48	62.88	-1.76
**Numerical Tests**	69.22	34.08	-11.1*

* Significant difference between younger and older adults, *p* <.05

### Materials

Two hundred and thirty six scene pictures from the International Affective Picture System (IAPS) were chosen on the basis of valence (positive 1–4, neutral 4–6 and negative 6–9) and arousal (positive 1–4, neutral 4–6 and negative 6–9) scores. These pictures were then rated for valence and arousal by 47 younger Chinese adults (26 women, 21 men, mean age = 23, range = 19–26 years), and 44 older Chinese adults (22 women, 22 men, mean age = 67, range = 60–78 years) using a 9-point scale. These raters did not take part in the eye movement and memory study reported in this paper.

We selected 28 positive, 28 negative, and 28 neutral pictures as experimental stimuli from those re-rated pictures. Independent samples t-tests and repeated measures ANOVA’s were used to compare the valence and arousal, and to compare between groups. There was no difference in ratings between younger and older people on all chosen pictures (*p*_*s*_ >. 05). Positive (5.40±1.04) and negative pictures (6.48±0.95) had higher arousal rating scores than neutral pictures (3.84±1.32), and did not differ from each other (*p* = .39). There were significant differences in valence between positive, negative and neutral pictures (*p*_*s*_ <. 05). The overall luminance levels of the pictures were slightly adjusted with the Adobe Photoshop 7.0 program to achieve uniform values for the different pictures. The means and standard deviations of the luminance level of each picture were computed with Scion Image software (Scion Corp, version Alpha 4.0.3.2, Frederick, Maryland). There were no significant differences in luminance level of all pictures (*p* = .673). The means and standard deviations of valence and arousal are shown in Table 2. The pictures were randomly assigned to form 14 pleasant-neutral pairs, 14 unpleasant-neutral pairs, and 14 pleasant-unpleasant pairs.

**Table 2:** Valence and Arousal Ratings for Positive, Negative, and Neutral Pictures.

	Younger adults			Older adults		
	Positive	Neutral	Negative	Positive	Neutral	Negative
**Valence**	6.48(0.69)	4.79(0.93)	2.55(0.61)	6.53(0.88)	4.87(0.95)	2.69(0.63)
**Arousal**	5.39(0.77)	4.07(1.12)	6.42(0.88)	5.41(1.31)	3.60(1.51)	6.54(1.02)

### Design

A mixed measures design was employed with a 3 (Pair Type): Type 1 (Pleasant versus Neutral); Type 2 (Unpleasant versus Neutral) and Type 3 (Pleasant versus Unpleasant) as within participants measures and a 1 (Group: Older versus Younger) as a between participant measure. This manipulation was similar to that used by Nummenmaa et al. [30]. Pair Types 1 and 2 allowed us to see whether there was evidence of an emotional processing bias when positive and negative pictures were paired with neutral pictures. Pair Type 3 was included as a more stringent test for the ‘Positive effect’ as this would allow a direct comparison as to whether older adults would ignore or avoid the unpleasant pictures when these were presented with a pleasant picture. Data were collapsed across block in our analyses to increase power, and on the basis that Nummenmaa et al. [30] had found no block differences for the emotional valence effects in their study.

### Procedure

**Fig 1:**
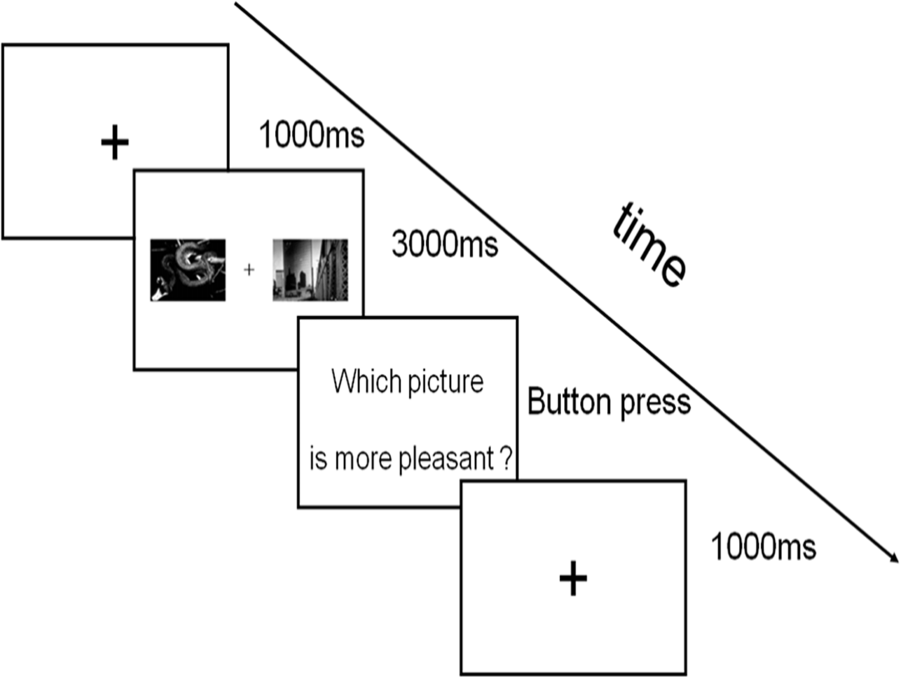
A schematic of the trial sequence.

An instruction sheet was given to participants that described what would happen in the experiment before the eye tracker was set up. A 9-point calibration procedure was then performed (with a requirement of maximum error of 0.05 degree of visual angle), followed by 8 practice trials and 42 experimental trials in each of two blocks. Fig 1 shows a schematic of the trial sequence. At the beginning of each trial, a “+” appeared at the centre of the screen for 1000ms, participants were asked to keep their eyes on the “+”. If the eyes deviated from the centre of the cross a recalibration was performed. The single “+” was followed by the presentation of two pictures (a pleasant, or an unpleasant picture paired with a neutral picture, or a pleasant paired with an unpleasant picture) on the screen, one on the left and the other on the right of the “+” and participants were instructed to look at the pictures. On one third of the trials participants had to indicate, using a button press, which of the two pictures was more pleasant. This manipulation was used to make sure that participants focused on the experiment and looked at both pictures during each trial. There was an interval of 1000ms between each trial. The same pictures were used in both blocks, but the two pictures that made up each trial display were reversed horizontally for block 2, and the whole experiment lasted about 20 minutes.

Following the eye movement experiment, both younger and older participants were given a recognition memory task for the pictures that were used in the experiment. They were instructed to respond ‘yes’ or ‘no’ and press the ‘F’ key if they had seen the picture in the previous experiment and the ‘J’ key if they had not seen the current picture. All 42 pictures from the eye movement study were included in the memory task, and 42 unseen pictures were added to these. The presentation order of the pictures from the eye movement task was counterbalanced across participants. We included a memory task to investigate whether participants were more likely to remember pleasant pictures over the other categories of picture, thus providing additional information in relation to any ‘Positive Effect’ that might be observed from the eye movement data. If participants are more likely to orient to and engage with the pleasant pictures then it would be expected that these pictures would be remembered more accurately.

### Data Collection

Participants’ eye movements were collected using an Eyelink eye-tracking system (SR Research Ltd., Canada) with a sampling frequency of 500Hz. Participants viewed the screen with both eyes but only the movements of the right eye were recorded. Pictures were presented on a 19-inch monitor with a refresh rate of 150 Hz and the monitor had a resolution of 1024 by 768 pixels. Participants were seated in a comfortable chair and the distance was 75cm away from the monitor. A chin rest stabilized head position. The pictures measured 10.4° by 7.2° of visual angle, with a distance of 3.2° of visual angle from the edge of each picture nearest to the screen centre to the actual screen centre. The interest areas were defined as each complete picture area on the left and right of the central “+”.

#### Data Analyses—T-tests

Since the picture pairings in the experiment were made up of different categories of pictures (from—Pleasant, Unpleasant and Neutral), it was not possible to analyse the mean scores for each category of picture in an ANOVA. We therefore computed a series of t-tests to compare the means for each category of picture that made up a pair of pictures, for both groups. We predicted that for the pairing of Pleasant with Neutral (Type 1)—if there was a positive effect then participants should orient faster to, and spend longer looking at the Pleasant picture. For the pairing of the Unpleasant picture with a Neutral picture (Type 2) we predicted that in order to support a ‘Positive Effect’ participants should avoid orienting to the negative image and should spend longer looking at the Neutral picture in the pair. When a Pleasant picture was paired with an Unpleasant picture then participants should orient to and remain looking longer at the Pleasant picture, to provide evidence for a ‘Positive Effect’.

#### Supplementary Data Analyses—Difference Scores for each Pair Type

A further analysis was conducted to enable us to compare whether any differences for the three picture pair conditions were equal across the three picture pair types, and for both groups. So, for all dependent measures we computed a difference score between the two types of picture that made up each trial display. For Pair Type 1 we subtracted the Neutral score from the Pleasant score to find the difference score. For Pair Type 2 we subtracted the Neutral score from the Unpleasant score to find the difference score. For Pair Type 3 we subtracted the Unpleasant from the Pleasant score to find the difference score. A repeated measures ANOVA with Pair Type (Type 1 versus Type 2 versus Type 3) as within participants variables, and age group (younger versus older) as a between participants variable was conducted on the absolute difference scores for the three dependent eye movement measures. The data files are available from the following link: http://thedata.harvard.edu/dvn/dv/positive/faces/study/StudyPage.xhtml?globalId=doi:10.7910/DVN/29002&studyListingIndex=2_f04b50b0058b26615178665ab588

## Results

The invalid data (including the data recorded when participants were moving their head and the data where participants were off centre at the beginning of the trials by more than one degree) were excluded (3.16% in total). We summarised the data from the interest areas by calculating the index of the probability of the first fixation landing in an interest area; the amount of time looking in that area before moving to the alternative picture (gaze duration) and the number of fixations made to an interest area from first fixating it and before leaving that area (first pass).

### T-test Results

#### Pleasant versus Neutral (Type 1)

If participants showed a ‘Positive Effect’ for this pairing then we would expect them to look at the Pleasant pictures for longer than the Neutral ones, and to be more likely to fixate pleasant pictures first.

#### The probability of first fixation

This measure is used to address any selective attentional orienting or preferences for one type of picture compared to another. The observed probability of first fixation (FF) landing in a picture is calculated by dividing the number of trials where the first fixation landed in the respective area of interest by the total trial numbers. The higher the probability the more attention is captured by that stimulus. There was no bias for early orienting as measured by the probability of first fixation landing in a picture. There were no effects for younger or older participants, both *t* ‘s < 2.

#### Gaze duration (GD)

Gaze duration is the sum of the duration of all fixations made from the first fixation within a region until a fixation is made outside this region. For both groups for Gaze Duration (GD) was significantly greater for the Pleasant picture: younger GD *t* (1, 26) = 7.29, *p* <. 001 (Pleasant 759ms vs Neutral 586ms); older GD *t* (1, 25) = 9.97, *p* <. 001 (Pleasant 687ms vs Neutral 500ms).

#### Number of First Pass Fixations (FP)

The number of the first-pass fixations is the count of all fixations within a region from the first fixation until a fixation is made outside this region. For both groups FP was significantly greater for the Pleasant picture: younger FP *t* (1, 26) = 2.32, *p* = .029 (Pleasant 2.62 vs Neutral 2.48); older FP *t* (1, 25) = 8.12, *p* <. 001 (Pleasant 2.6 vs Neutral 1.93).

#### Unpleasant versus Neutral (Type 2)

If participants showed a ‘Positive Effect’ for this pairing then we would expect them to avoid looking at the unpleasant pictures and to look for longer at the neutral pictures. This was not what we observed.

#### The probability of first fixation

For this early orienting measure for both groups the first fixation probability was greater for the unpleasant compared to the neutral pictures, younger FF *t* (1, 26) = 7.47, *p* <. 001 (Unpleasant 52.78 vs Neutral 31.61); older FF *t* (1, 25) = 3.63, *p* = .001 (Unpleasant 47.78 vs Neutral 27.75), In contrast to the overall finding for the analysis for the Pleasant and Neutral pair, where there were no effects of early orienting—for the Unpleasant and Neutral pair both groups showed an early orienting effect and were more likely to first fixate the Unpleasant compared to the Neutral picture.

#### Gaze duration (GD)

For both groups there were no differences in Gaze Duration between the unpleasant and neutral pictures, younger and older, both GD *t’s < 2*.

#### Number of First Pass Fixations (FP)

For both groups there were no differences in First Pass Fixations between the unpleasant and neutral pictures, younger and older FP both *t’s < 2*.

#### Pleasant versus Unpleasant (Type 3)

If participants showed a ‘Positive Effect’ for this pairing then we would expect them to avoid looking at the unpleasant pictures and be more likely to fixate the pleasant pictures first, and to look for longer at the pleasant compared to the unpleasant pictures.

#### The probability of first fixation

For both groups the first fixation probability was greater for the pleasant compared to the unpleasant picture, younger FF *t* (1, 26) = 2.52, *p* = .018 (pleasant 45.50 vs unpleasant 37.30); older FF *t* (1, 25) = 3.39, *p* = .002 (pleasant 40.94 vs unpleasant 29.53).

#### Gaze duration (GD)

For the younger group for there was no difference in Gaze Duration between the pleasant and unpleasant pictures, younger GD *t* < 2. In contrast the older group spent more time looking at the pleasant pictures before moving to the unpleasant ones *t* (1, 25) = 3.41, *p* = .002 (pleasant 608ms vs unpleasant 544ms).

#### Number of First Pass Fixations (FP)

**Fig 2:**
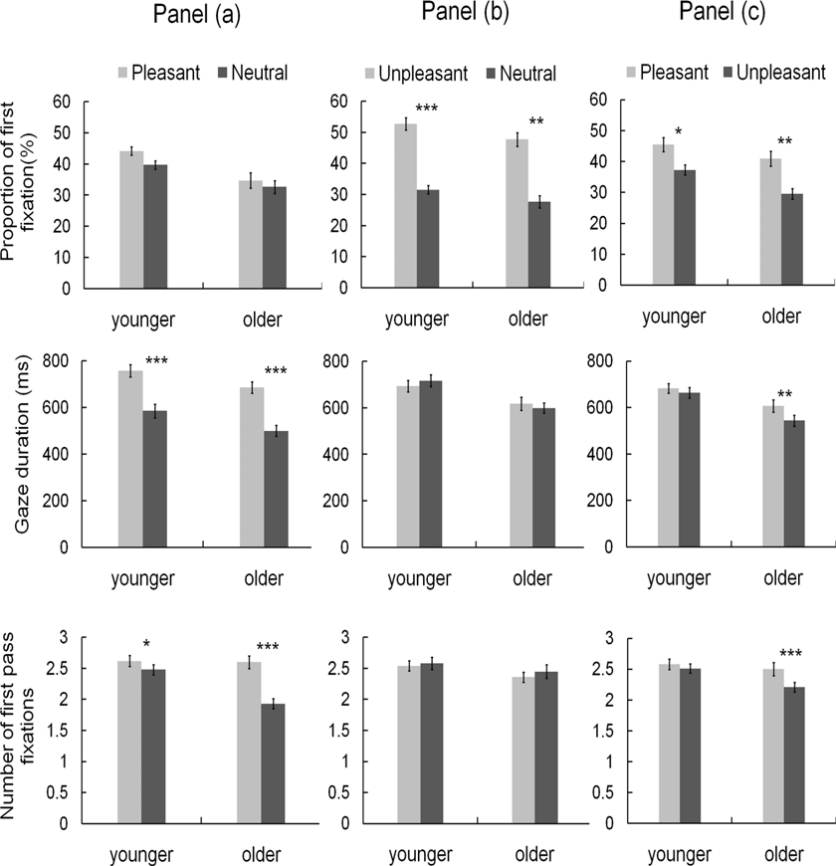
The means for the three different eye movement measures, for the three different picture pairings, for the older and younger participants are shown in Fig 2. Panel (a) presents the data for the pleasant and neutral picture pairs; Panel (b) presents the data for the unpleasant and neutral picture pairs; Panel (c) presents the data for the pleasant and unpleasant picture pairs. Significance levels are represented as follows—* *p* <.05, ** *p* <.01, ***p <.001.

For the younger group for there was no difference in First Pass Fixations between the pleasant and unpleasant pictures, younger FP *t* < 2. Similar to the findings for GD the older group had a greater number of fixations to the pleasant pictures before moving to the unpleasant ones *t* (1, 25) = 4.54, *p* <. 001 (pleasant 2.50 vs unpleasant 2.21). Fig 2 shows the eye movement measures for both groups and all three pairings.

### Summary

The overall findings from the t-tests have shown that there is an emotional bias for both groups—but that this bias is reflected in different ways. Initial orienting effects (first fixation on target) are observed when unpleasant pictures are paired with neutral pictures, whereas initial orienting effects are absent when pleasant and neutral pictures are paired. However, in that condition, engagement effects, as measured by the number of first pass fixations and gaze duration, are observed for both groups who spend longer inspecting the pleasant pictures before moving the eyes to a different location. The combination of pleasant and unpleasant pictures allowed us to look at avoidance of negative information and preferential engagement with positive emotional information when these two types of emotional information were presented together. Here, and in contrast to the previous finding, both groups showed an initial orienting preference for pleasant over unpleasant pictures, and furthermore the older group also showed a bias for the positive pictures in the later measures too—indicating strong evidence of a ‘Positive Effect’ in that group.

#### Difference Scores Analysis

**The probability of first fixation**. Difference scores analyses show that, the main effect of Type was significant, *F* (2, 102) = 6.25, *p* = .003. Type1 (9.99) was smaller than Type2 (17.23) and Type3 (14.90). When a pleasant picture is paired with an unpleasant picture, and when an unpleasant picture is paired with a neutral picture initial orienting to one type of picture over another is greater than that shown for Type 1 picture pairs. This main effect was qualified by an interaction between Type and Group, *F* (2, 102) = 5.73, *p* <. 05 which showed that the probability of fixating one type of picture over another was greater for older participants compared to younger participants for Type 1 (younger 8.04, older 11.95), *p* = .046, whereas this effect was greater for younger participants compared to older participants for Type 2 (younger 21.96, older 12.50), *p* = .009. For Type 3, there was no group difference (younger 14.97, older 15.52), *p* = .724. The interaction demonstrates that there’s no group difference for Type 3, but younger show a bigger effect for Type 2 where unpleasant pictures are paired with neutral, whereas the older show a bigger effect for Type 1, where pleasant pictures are paired with neutral.

#### Gaze duration (GD)

Difference scores show that, the main effect of Type is significant, *F* (2, 102) = 32.82, *p* <. 001. Type1 (187) was greater than Type2 (95), and Type1 (187) was also greater than Type3 (81). There were no other effects.

#### Number of First Pass Fixations (FP)

Difference scores showed that, the main effect of Type was significant, *F* (2, 102) = 24.12, *p* <. 001. Type1 (0.65) was greater than Type2 (0.27), and Type1 (0.65) was also greater than Type3 (0.28). There were no other effects.

### Summary

The difference scores analyses provides additional information concerning initial orienting to and processing of emotional pictures, and it shows that there are bigger effects for one type of pairing over another, and the nature of the effects (for initial orienting or later processing) is also influenced by picture pair type.

### Memory for the Pictures

In previous studies on the ‘Positive Effect’, both attention and memory have been examined and both have been shown to reflect the ‘Positive Effect’ in older participants in western societies. We therefore also tested for recognition accuracy for the pictures that had been presented in the eye movement study. Accuracy was calculated as the proportion of new pictures identified as not having been previously seen, and the proportion of pictures that had been seen previously being identified as such.

We found a significant main effect of picture valence (*F*(2,96) = 21.03, *p* = .002). Post hoc tests showed that participants remembered more pleasant pictures than unpleasant (*p* = .032 and neutral ones (*p* = .044), and the accuracy of unpleasant pictures was not significantly different from the neutral ones (*p* = .073). The main effect of age group was also significant (*F*(1,48) = 6.33, *p* = .004), which revealed that the younger adults kept more pictures in mind than the older adults. There was no interaction between these two variables (*F*(1,48) = 0.68, *p* = .851). Table 3 shows the means and standard deviations for the recognition accuracy for the younger and older participants. As shown in Table 3, both younger and older participants showed a similar pattern to recognize more pleasant pictures than neutral and unpleasant ones.

**Table 3:** Recognition Accuracy for the Pictures.

	Positive		Negative		Neutral	
	*M*	*SD*	*M*	*SD*	*M*	*SD*
**Older**	0.81	0.11	0.75	0.13	0.71	0.11
**Younger**	0.88	0.13	0.83	0.13	0.80	0.12

## Discussion

The present study investigated whether there was a ‘Positive Effect’ in Chinese participants from an East Asian culture. We found that Chinese elders did show a ‘Positive Effect’, and both, younger and older Chinese participants showed an emotional bias for both pleasant and unpleasant pictures when these were paired with a neutral picture. When pleasant pictures were paired with unpleasant pictures, both groups showed a preference to initially orient to the pleasant picture but both types of picture were looked at for the same amount of time in the younger group, whereas the older group showed a bias to engage with the pleasant pictures for longer. For the memory test both groups remembered more positive than negative pictures.

The results are inconsistent with Fung et al.’s study [18], which suggested that there was no ‘Positive Effect’ in an older population, at least not in the Chinese culture. However, it has also been argued that residents in Hong Kong who took part in Fung et al.’s experiment were not typical representatives of individuals in East Asian culture [31]. It was pointed out that Hong Kong is a colony that was controlled by Britain for more than a century, therefore, Hong Kong residents have been unavoidably influenced by Western culture. The participants who took part in the current study were from mainland China, (the city of Tianjin), thus they were considered to be appropriate representatives of people from an East Asian culture. It has also been suggested that the lack of a ‘Positive Effect’ in Fung et al.’s study resulted from the use of synthetic faces in that study, which may not reflect emotion expressed in real faces, and nor would they reflect the complexity present in emotional scenes. The scene pictures chosen from the IAPS that were used in our study allowed us to avoid such a potential confound. Kwon and colleagues [24] found a ‘Positive Effect’ in older Koreans, but this was for memory, and they did not observe or analyse how attention was allocated to the pictures. Memory processes do not equal attention processes, therefore, it cannot be deduced directly that older populations in East Asian pay more attention to positive stimuli from Kwon’s study.

In the current study, compared with neutral pictures, participants looked at pleasant emotional pictures for longer time (longer gaze duration) and had more fixations (larger number of first-pass fixations) within the pleasant picture areas before they moved their eyes to other regions on the screen. A different bias was observed when unpleasant emotional pictures were presented with neutral pictures. Here participants were more likely to fixate the unpleasant picture first, but then showed no preference in engagement with the two types of pictures presented in that condition. Importantly, those eye movement measures showed no differences between positive and negative pictures not only for younger participants but also for older participants. If we had only these two picture combinations to compare, then we would conclude that both groups had an emotional bias, and that this would be reflected either in early orienting, or later processing, depending on whether it was positive or negative information that was presented with neutral information However, an important difference that was observed when unpleasant pictures were paired with pleasant pictures showed that initially, both groups oriented to the pleasant, and not the unpleasant picture, as they had done when unpleasant pictures were paired with neutral pictures; and additionally, the older group showed a preference for attending to positive emotional information for longer.

These findings are novel and demonstrate that whether an emotional bias is present for early orienting measures, or later processing measures, is dependent upon whether the emotional information is positive or negative. Furthermore initial orienting effects to unpleasant emotional information can be eliminated when unpleasant information is paired with pleasant information—something that has not previously been reported. It should though be noted that, the initial orienting to the pleasant over the unpleasant pictures could possibly have been influenced by the effect of the instruction in the current study, where the task was to say which picture was more pleasant on a proportion of the trials—a neutral instruction should be used for future studies.

Our evidence for the presence of a ‘Positive Effect’ in older Chinese participants was strongest when pleasant information was presented simultaneously with unpleasant information, and here we observed avoidance of the negative and preference for the positive in both early and late processing measures—a finding which reflects both facets of the ‘Positive Effect’.

Our findings contradict those theories that posit that in Eastern cultures suppression of positive self-expression and a focus on self-criticism could lead to preferential attention to negative rather than positive information, and that this effect would be long lasting in Chinese cultures [18]. Overall whilst we cannot conclude that the data support the SST theory [15] we can say that older Chinese adults, similar to older Western adults, prefer to focus on positive emotional information. However, in order to determine the existence and nature of any true cross cultural differences in biases for one type of emotional information over another, we aim to carry out a cross cultural experiment to directly test both Eastern and Western cultures ability to show a 'Positive Effect', using the same pictures and paradigm.

In conclusion, older participants showed the same attentional bias as younger participants to both positive and negative pictures when these were paired with neutral pictures. However when positive and negative pictures were presented together, a ‘Positive Effect’ was observed exclusively for the older group, suggesting that Chinese people do prefer to focus on the positive, especially when positive information is presented along side negative information.
